# Porcine Pancreatic Lipase Inhibitory Agent Isolated from Medicinal Herb and Inhibition Kinetics of Extracts from* Eleusine indica* (L.) Gaertner

**DOI:** 10.1155/2016/8764274

**Published:** 2016-10-31

**Authors:** Siew Ling Ong, Siau Hui Mah, How Yee Lai

**Affiliations:** School of Biosciences, Taylor's University, No. 1 Jalan Taylor's, 47500 Subang Jaya, Malaysia

## Abstract

*Eleusine indica* (Linnaeus) Gaertner is a traditional herb known to be depurative, febrifuge, and diuretic and has been reported with the highest inhibitory activity against porcine pancreatic lipase (PPL) among thirty two plants screened in an earlier study. This study aims to isolate and identify the active components that may possess high potential as an antiobesity agent. Of the screened solvent fractions of* E. indica*, hexane fraction showed the highest inhibitory activity of 27.01 ± 5.68% at 100 *μ*g/mL. Bioactivity-guided isolation afforded three compounds from the hexane fraction of* E. indica*, namely, *β*-sitosterol, stigmasterol, and lutein. The structures of these compounds were elucidated using spectral techniques. Lutein showed an outstanding inhibitory activity against PPL (55.98 ± 1.04%), with activity 60% higher than that of the reference drug Orlistat. The other compounds isolated and identified were *β*-sitosterol (2.99 ± 0.80%) and stigmasterol (2.68 ± 0.38%). The enzyme kinetics of* E. indica* crude methanolic extract on PPL showed mixed inhibition mechanism.

## 1. Introduction

Obesity is often defined as the excess accumulation of body fat resulting from a higher energy intake than energy expenditure [1]. In 2008, 10% of men and 14% of women in the world were obese, compared with 5% of men and 8% of women in 1980 [2]. Rates of both overweight and obesity are projected to increase in almost all countries, with 1.5 billion people overweight in 2015 [2]. Pancreatic lipase inhibition is one of the most widely studied mechanisms for antiobesity treatment, based on the principle that dietary fat will not be directly absorbed by the intestine unless the fat has been subjected to the action of pancreatic lipase [3, 4].

Phytochemicals or bioactive compound/extract identified from traditional medicinal plants had provided an exciting platform and opportunity for the development of safe and effective therapeutic drugs for the treatment of many metabolic diseases [5]. A review by Newman and Cragg (2007) [6] on the origin of drugs launched in the past 25 years showed about half of the compounds that were successful in clinical trials were derived from natural origin. Despite multiple research conducted in recent decades, the potential of antiobesity therapeutic drug of natural product origin is still largely unexplored. Previous screening study on thirty two plants reported strongest porcine pancreatic lipase (PPL) activity in* E. indica* [7] and this has led to further investigation on this herb for potential antiobesity agent.

*E. indica* (Linnaeus) Gaertner (Poaceae) is an annual grass native in the tropics and subtropical regions [8, 9]. It is commonly widespread as weed in rice field and is known to be resistant to many herbicides (such as dinitroaniline) [10]. This plant is commonly known as goosegrass, wiregrass, “rumput sambari,” or “rumput sambau” in Malaysia [11]. Its root is traditionally known to be depurative, febrifuge, diuretic, and laxative and thus is commonly used for treating hypertension, influenza, oliguria, and urine retention [8]. The decoctions of the boiled whole plant are consumed for antihelminthic and febrifuge treatment [12]. The seed of* E. indica* is sometimes used as famine food and in the treatment for liver complaints [13].

Several pharmacological properties on* E. indica* have been reported including hepatoprotective effect [13], antiplasmodial and antidiabetic [14], antioxidant and antimicrobial activity [8], anti-inflammatory [15], and cytotoxic effect towards several cancer cell lines [8, 16]. To date, only one study reported the isolation of secondary metabolites from* E. indica* where hexadecanoic acid and [[(2-aminoethoxy) hydroxyphosphinyloxy]methyl]-1,2–ethanediylester were isolated [17]. Hence, this paper is the first report on the kinetics of PPL enzyme inhibition by* E. indica* and the bioactivity-guided isolation of a potent PPL inhibitory compound (lutein) from* E. indica*.

## 2. Materials and Methods

### 2.1. Plant Materials, Extraction, and Preparation of Crude Extracts

The whole plants of* E. indica* (L.) Gaertn. were collected from Persatuan Pengkaji Herba Tradisional Negeri Sembilan (Pantai, Negeri Sembilan, coordinates: 2°46′13′′N, 101°59′40′′E). This plant was authenticated by Dr. Fadzureena Jamaludin from Forest Research Institute Malaysia (FRIM); the voucher specimen 003/15 (collection date: 11 February 2015) is kept at the School of Biosciences, Taylor's University (Lakeside Campus).

The whole plant of* E. indica* was cleaned from residual soil, freeze-dried, and pulverised. Analytical grade methanol was added and the extracts were then filtered and pooled, and the solvent was evaporated off.

### 2.2. Subextraction of the Main Extract

The crude extract of* E. indica* was suspended in distilled water (1 : 10, w/v) and sequentially extracted with solvents in increasing polarity (hexane, chloroform, ethyl acetate, and butanol), three times each (1 : 1, v/v), to obtain the respective solvent fractions. Each fraction was then assayed for porcine pancreatic lipase inhibition activity.

### 2.3. Porcine Pancreatic Lipase (PPL) Inhibition Assay

Porcine pancreatic lipase (PPL) inhibitory assay was performed as described by Bustanji et al. (2011) [18] with minor modification. The enzyme solutions was prepared immediately before use, by suspending crude porcine pancreatic lipase powder type II (Sigma, EC 3.1.1.3) in Tris-HCl buffer (50 mM Tris, 150 mM NaCl, 1 mM EDTA, 10 mM MOPS, pH 7.6) to give a concentration of 5 mg/mL (200 units/mL). The solution was then centrifuged at 1,500 rpm for 10 minutes and the clear supernatant was recovered. The plant extract (100 *μ*g/mL) was preincubated with 200 *μ*L of PPL solution for 5 minutes at 37°C, before the addition of 5 *μ*L PNPB substrate solution (10 mM in acetonitrile). The total reaction volume was made to 1 mL using the Tris-HCl buffer before measuring the absorbance at 410 nm against blank using denatured enzyme. The denatured enzyme was prepared by boiling the enzyme solution for 5 minutes. Orlistat was used as a reference drug. The extract was dissolved in DMSO at a final concentration not exceeding 1% (v/v) which will not affect enzyme activity.

The activity of the negative control was checked with and without the inhibitor. The inhibitory activity (*I*%) was calculated according to the formula below [18]:

(1) $$\begin{array}{c} I\%=\left(\;1-\frac{B-b}{A-a}\;\right)\times 100, \end{array}$$

where* A* is the activity of the enzyme without inhibitor,* a* is the negative control without the inhibitor,* B* is the activity of the enzyme with inhibitor, and* b* is the negative control with inhibitor.

### 2.4. Kinetic Study

The inhibition mode of* E. indica* methanolic crude extract on porcine pancreatic lipase (PPL) was assayed with increasing concentrations (20, 40, 60, and 80 *μ*M) of synthetic substrate,* p*-nitrophenyl butyrate (PNPB), in the presence and absence of two different concentrations of the extracts (100 and 200 *μ*g/mL). The mode of inhibition was determined by Lineweaver-Burk plot of the data.

### 2.5. Chromatography and Spectral Instrumentation

#### 2.5.1. Thin Layer Chromatography (TLC)

The TLC was performed on the TLC Silica Gel 60 coated with fluorescent indicator F_254_ Aluminium sheets (Merck). Samples were spotted and viewed under UV lamp at 254 nm and 365 nm.

#### 2.5.2. High Performance Liquid Chromatography (HPLC)

The fingerprinting of the extracts was done on Shimadzu Prominence Series coupled with photodiode array (PDA) detector SPD-M20A using either reversed-phase or normal phase settings:

- Reversed-phase, Chromolith HighResolution RP-18 endcapped 100–4.6 mm (Merck): the mobile phase used was solvent A, acetonitrile and solvent B, water with a standard flow rate of 1.0 mL/min, and injection volume of 20 *μ*L of 10 mg/mL extract; the gradient of the mobile phase was as follows: 0% to 50% A (0–45 min).
- Normal phase, Phenomenex Luna Silica column (250 × 4.6 mm, 100 Å, 5 *μ*m): the mobile phase used consists of solvent A, hexane and solvent B, 2-propanol with a flow rate of 1.0 mL/min, and injection volume of 20 *μ*L of 10 mg/mL extract; the gradient program was as follows: 100% A (0–5 min), 100% to 0% A (5–25 min).

#### 2.5.3. Infrared Spectroscopy (FT-IR)

The IR spectra were measured by Perkin Elmer Spectrum 100 using potassium bromide pellet method.

#### 2.5.4. Gas Chromatography–Mass Spectrometry Using Electron Impact Ionisation (GC-EI-MS)

Mass spectra were recorded with EIMS using a Direct Injection Probe on a Shidmadzu GC-MS QP 5050A Spectrometer. GC-MS was performed to identify the purity and molecular weight of compounds.

#### 2.5.5. UV-Visible Spectra

The UV-Vis spectra were recorded on a Thermo Scientific Genesys 10 UV Scanning Spectrophotometer.

#### 2.5.6. Melting Point

Melting points were recorded using a melting point probe Electrothermal IA 9000 Series.

#### 2.5.7. Nuclear Magnetic Resonance (NMR)

The spectra were obtained from JEOL ECX500 FT NMR Spectrometer system. Deuterated chloroform (CDCl_3_) was used as the solvent to dissolve the test samples. Tetramethylsilane (TMS) was used as internal standard for both ^1^H (500 MHz) and ^13^C (125 MHz). The chemical shifts from the spectra were recorded in ppm and coupling constants were given in Hertz (Hz).

### 2.6. Bioactivity-Guided Extraction and Isolation

**Figure 1:**
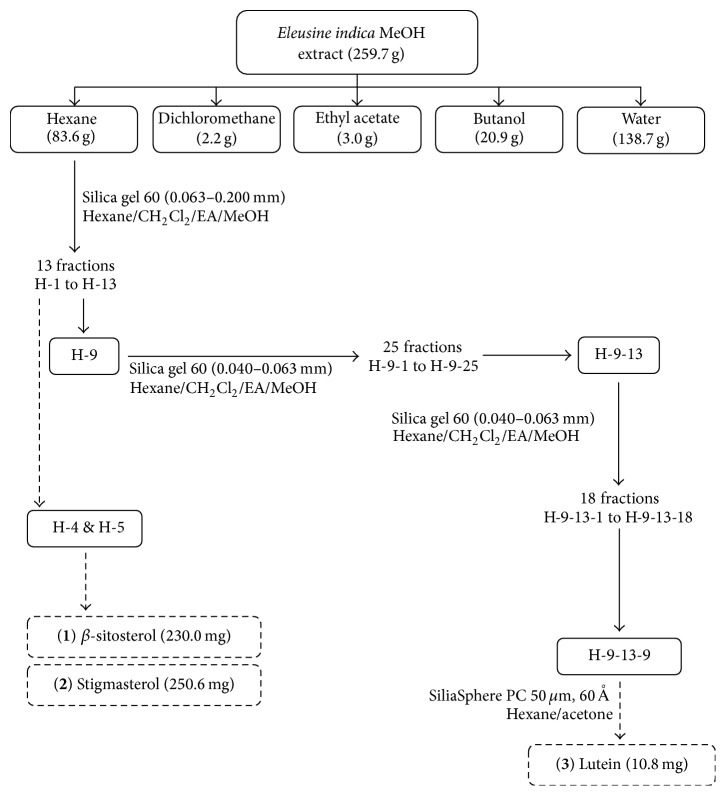
Schematic flow of the bioactivity-guided isolation on* E. indica* extract.

The active fraction was subjected to gravitational column chromatography packed with suitable packing materials; the schematic flow is as shown in Figure 1. The weight of the selected packing materials introduced into the column was at least ten times the weight of the sample extract. Sample was separated using the solvent system as stated in Figure 1.

The isolated pure compounds were then characterised and elucidated employing several spectral methods as stated in Section 2.5.

*β-sitosterol* (**1**) White crystal; m.p. 134.5–137.6°C; UV (Hexane) *λ*_max_ nm (log *ε*): 210 (817), 230 (54); IR *υ*_max_ cm^−1^: 3431, 2937, 1468, 1382, 1056; EIMS* m/z* (rel. int.): 414 [M^+^], 329 (20), 145 (25), 107 (30), 105 (27), 91 (20), 93 (21), 95 (28), 81 (28), 69 (27), 57 (51), 55 (36), 43 (100), 41 (32); ^1^H NMR (500 MHz, CDCl_3_): *δ* 5.29 (m, 1H, H-3), 3.46 (m, 1H, H-6); ^13^C NMR (125 MHz, CDCl_3_): *δ* 140.7 (C-5), 121.8 (C-6), 79.1 (C-3 & C-13), 55.4 (C-14 & C-17), 50.6 (C-9), 48.7 (C-24), 42.1 (C-4), 39.5 (C-12), 39.0 (C-1), 38.4 (C-10), 37.2 (C-20), 33.4 (C-22), 32.7 (C-2), 31.2 (C-7 & C-8), 29.8 (C-25), 27.5 (C-16), 26.8 (C-23), 26.3 (C-15), 25.6 (C-28), 21.5 (C-11), 19.6 (C-26), 18.4 (C-19 & C-27), 17.8 (C-21), 15.5 (C-29), 14.9 (C-18).

*Stigmasterol* (**2**) White crystal; m.p. 168.0–170.0°C; UV (Hexane) *λ*_max_ nm (log *ε*): 210 (839), 230 (147); IR *υ*_max_ cm^−1^: 3426, 2936, 1645, 1465, 1384, 1052 and 959; EIMS* m/z* (rel. int.): 412 [M^+^] (61), 255 (52), 159 (56), 145 (61), 95 (58), 81 (78), 69 (65), 55 (100); ^1^H-NMR (500 MHz, CDCl_3_): *δ* 5.34 (d, 1H,* J* = 4.6 Hz, H-6), 5.16 (m, 1H, H-22), 5.03 (m, 1H, H-23), 3.53 (m, 1H, H-3), 1.00 (s, 3H, H-19), 0.916 (d, 1H,* J* = 5.75 Hz, H-21), 0.829 (m, 9H, H-26, H-27, H-29), 0.685 (s, 3H, H-18); ^13^C NMR (125 MHz, CDCl_3_): *δ* 140.8 (C-5), 138.4 (C-20), 129.4 (C-21), 121.8 (C-6), 71.9 (C-3), 56.9 (C-14), 56.2 (C-17), 51.3 (C-22), 50.2 (C-9), 42.4 (C-4), 42.3 (C-13), 39.9 (C-12), 39.8 (C-18), 37.4 (C-1), 36.6 (C-10), 32.0 (C-2), 31.7 (C-7), 29.2 (C-8), 29.0 (C-16), 28.3 (C-25), 25.5 (C-23), 24.4 (C-15), 21.2 (C-11), 19.9 (C-27), 19.5 (C-26), 19.1 (C-19), 18.9 (C-28), 12.3 (C-29), 12.1 (C-24).

*Lutein* (**3**) orange crystal; m.p. 173.8–174.9°C; UV (Acetone) *λ*_max_ nm (log *ε*): 266, 426 (shoulder), 448 (628), 476 (564); IR *υ*_max_ cm^−1^: 3427, 2926, 1718, 1465, 1376, 1261; EIMS* m/z* (rel. int.): 568 [M^+^], 145 (49), 119 (96), 105 (100), 93 (54), 91 (70); ^1^H NMR (500 MHz, CDCl_3_): *δ* 6.57 (m, 4H, H-11, H-15, H-11′, H-12′), 6.34 (d, 2H,* J* = 14.9 Hz, H-12), 6.23 (m, 2H, H-14, H-14′), 6.10 (m, 5H, H-8, H-10, H-7′, H-8′), 5.54 (s, 1H, H-4), 5.39 (dd, 1H,* J* = 10.4, 9.2 Hz, H-7), 4.24 (s, 1H, H-3), 3.98 (m, 1H, H-3′), 2.36 (m, 2H, H-6, H-4eq′), 2.01 (dd, 1H,* J* = 10.3, 9.2 Hz, H-4ax′), 1.96 (s, 9H, H-20, H-19′, H-20′), 1.90 (s, 3H, H-19), 1.81 (dd, 1H,* J* = 5.7, 6.9 Hz, H-2eq), 1.73 (s, 3H, H-18′), 1.62 (s, 12H, H-18), 1.44 (t, 1H,* J* = 12.6, 11.5 Hz, H-2′), 1.34 (dd, 1H,* J* = 5.7, 6.9 Hz, H-2ax), 1.06 (s, 6H, H-16′, H-17′), 0.838 & 0.987 (s, 6H, H-16, H-17); ^13^C NMR (125 MHz, CDCl_3_): *δ* 138.6 (C-8′), 138.1 (C-8), 137.8 (C-5, C-12′), 137.6 (C-12, C-6′), 136.6 (C-13′), 136.5 (C-13), 135.8 (C-9′), 135.2 (C-9), 132.7 (C-14, C-14′), 131.4 (C-10′), 130.9 (C-10), 130.2 (C-15′), 130.1 (C-15), 128.8 (C-7), 126.2 (C-5′), 125.7 (C-7′), 125.0 (C-4), 124.9 (C-11′), 124.6 (C-11), 66.0 (C-3), 65.2 (C-3′), 55.1 (C-6), 48.5 (C-2′), 44.7 (C-2), 42.6 (C-4′), 37.2 (C-1′), 34.1 (C-1), 30.3 (C-17′), 29.6 (C-17), 28.8 (C-16′), 24.4 (C-16), 22.8 (C-18), 21.7 (C-18′), 13.2 (C-19), 12.9 (C-19′, C-20), 12.8 (C-20).

### 2.7. Statistical Analysis

All results were expressed as mean ± standard deviation. Significance of difference from the control was determined by Tukey's* post-hoc* test (one-way ANOVA)* p* value < 0.05 using SPSS software (version 16.0).

## 3. Results and Discussion

### 3.1. Kinetic Analysis

**Figure 2:**
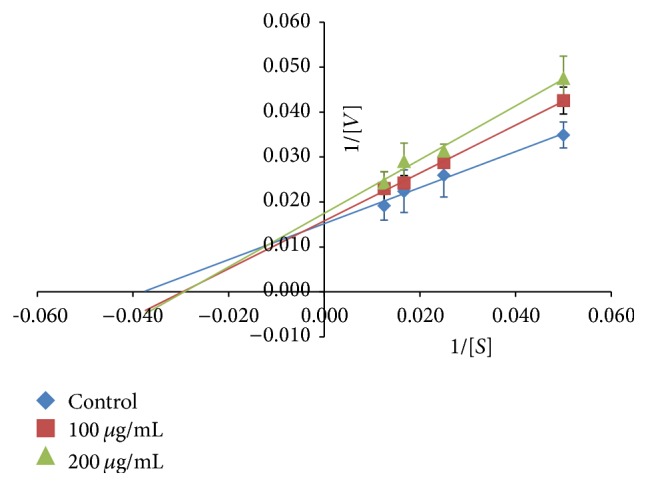
Lineweaver-Burk plot of* E. indica* methanolic extract against PPL at two different concentrations (100 and 200 *μ*g/mL). Bar indicates the standard deviation.

The inhibition mode of PPL by* E. indica* methanolic extract at 100 *μ*g/mL and 200 *μ*g/mL was analysed by double-reciprocal Lineweaver-Burk plot as shown in Figure 2. Kinetic parameters calculated from the double reciprocal trend lines showed that both the maximal velocity of the PPL enzyme-substrate extract reaction (*V*_max_) and the affinity (*K*_m_) were affected by the extract concentration, hence indicating a mixed mode inhibition. The Michaelis-Menten parameters are tabulated in Table 1, where the Michaelis-Menten constant (*K*_m_) of PPL with synthetic substrate PNPB was 26.36 *μ*M and maximal velocity (*V*_max_) was 65.79 *μ*M min^−1^. The mixed mode inhibition exhibited by PPL indicates that the formation of enzyme-substrate complex was possible with the inhibitor binding at a distinct site from the active site resulting in reduction in the complex affinity, thus explaining the increase in *K*_m_. Similar inhibition mode was observed in* Levisticum officinale* methanolic extract and regular cocoa extract against porcine pancreatic lipase [19, 20].

The methanolic crude extract of* E. indica* was then partitioned via liquid-liquid fractionation, to yield five (5) solvent extracts, that is, hexane, dichloromethane, ethyl acetate, butanol, and water. All solvent extracts were then assayed for their inhibitory activity against PPL.  Table 2 shows that the hexane extract from* E. indica* possessed the highest PPL inhibitory activity, that is, 27.01 ± 5.68%. Although* E. indica* dichloromethane extract recorded a comparable value of 25.57 ± 3.26% PPL inhibitory activity, due to its low yield from the partition (1.18%), this extract was not further tested.

### 3.2. Fingerprinting of Solvent Extracts from* E. indica*

**Figure 3:**
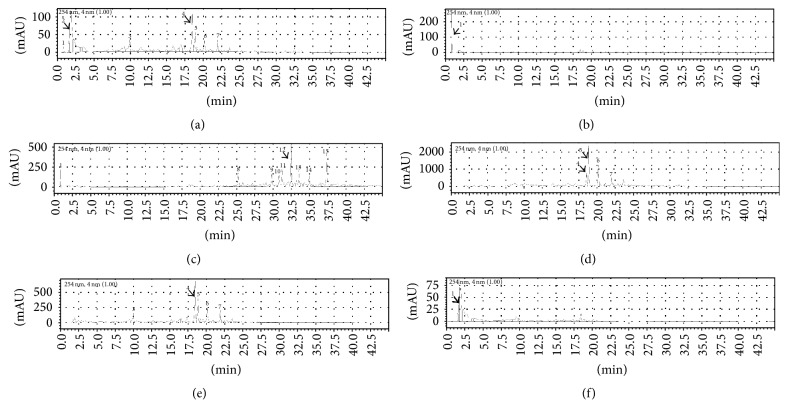
HPLC profiles of different fractions of* E. indica*: (a) crude methanolic extract; (b) hexane fraction; (c) dichloromethane fraction; (d) ethyl acetate fraction; (e) butanol fraction and; (f) aqueous fraction using Chromolith HighResolution RP-18 (Merck) HPLC column (reverse phase) at 254 nm using the gradient mobile phase 0% to 50% acetonitrile: 100% to 50% deionised water (0 to 45 minutes).

**Figure 4:**
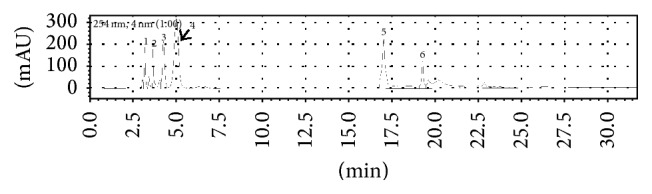
HPLC profiles of* E. indica* hexane fraction using Luna 5 *μ*m Silica (Phenomenex) HPLC column (normal phase) at 254 nm using the gradient mobile phase 100% hexane for 5 minutes, followed by 100% to 0% of hexane: 0% to 100% 2-propanol (5 to 25 minutes).

The HPLC chromatograms of all* E. indica* methanolic extract and solvent fractions are shown in Figure 3. Six major peaks were detected in elution profile of the crude methanolic extract of* E. indica* (Figure 3(a)). No peak was detected in hexane fraction (Figure 3(b)) due to the incompatibility of reverse phase column with the nature of the fraction, which was highly nonpolar. The hexane fraction was later optimised and run with Luna 5 *μ*m Silica (Phenomenex), a normal phase column coupled with normal phase mobile phase, where six major peaks were detected as shown in Figure 4. A cluster of eight peaks were eluted in the second half end of the chromatogram in dichloromethane fraction (Figure 3(c)) due to its more nonpolar nature. Most of the peaks from this cluster (peak 8 to peak 15) were visible in the chromatogram of crude methanolic extract in Figure 3(a), but the concentration of these compound present in the crude extract was too low to be detected as the major peaks. Figures 3(d) and 3(e) which showed the elution profile of ethyl acetate and butanol extracts, respectively, recorded peak 4 to peak 7 which corresponded to the major peaks as detected in elution profile of crude methanolic extract where they represented elution of semipolar compounds at the midrange of the chromatograms. The most polar compounds were eluted at the beginning of the spectrum (retention time less than 2.5 minutes) in the aqueous fraction (Figure 3(f)), which were also detected as one of the major peaks in the crude extract.

High PPL inhibitory activity was detected in hexane (27.01 ± 5.68%) and dichloromethane (25.57 ± 3.26%) fractions from* E. indica* (Table 2). Chromatogram of the hexane fraction (Figure 4) showed presence of six (6) major components (numbers 1 to 6) while chromatogram of the dichloromethane fraction (Figure 3(c)) showed eight major components (numbers 8 to 15). Attempts were made to identify the components by running with ten standards, namely, rutin, quercetin, neringenin, caffeic acid, chlorogenic acid, coumaric acid, gallic acid, esculin, kaempferol, and myricetin. However, none of the peaks' retention time matched those of the standards. As such, the components eluted from the hexane and dichloromethane fractions could not be identified from the chromatograms. Nevertheless, these chromatographic fingerprints obtained from the methanolic crude and all solvent fractions may provide a simple guide for future identification and comparison of* E. indica* L. even if the geographical location and/or season of collection are different.

### 3.3. Bioactivity-Guided Extraction

**Figure 5:**
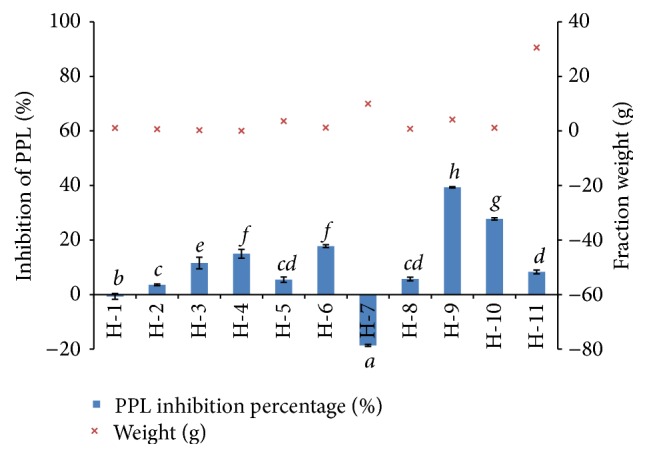
The fraction yield and PPL inhibitory activity of H-1 to H-11. Bar indicates the standard deviation. Statistically significant effects were compared using one-way ANOVA (Tukey's* post-hoc* test), where different letters indicated significance at *p* < 0.05.

The hexane fraction of* E. indica* was further separated into eleven (11) fractions.  Figure 5 shows H-9 (4.21 g) recorded the highest PPL inhibitory activity, that is, 39.35 ± 0.24%, which was obtained from the elution with 75% dichloromethane/25% ethyl acetate. The remaining fractions obtained from this column recorded less than 20% of PPL inhibitory activity.

Alongside with the separation of H-1 to H-11, two common sterols were isolated from the fractions H-4 and H-5, on elution with 90% dichloromethane/10% ethyl acetate. *β*-sitosterol (**1**) was isolated as a white flower-liked crystalline powder while stigmasterol (**2**) was in white needle-like crystals. The detailed physical properties of *β*-sitosterol and stigmasterol will be discussed in the later part of this paper. Both compounds were found to possess very low PPL inhibition activity, that is, 2.99 ± 0.80% (*β*-sitosterol) of inhibition at 100 *μ*g/mL (242 *μ*M) and 2.68 ± 0.38% (stigmasterol) of inhibition at 100 *μ*g/mL (243 *μ*M), respectively.

**Figure 6:**
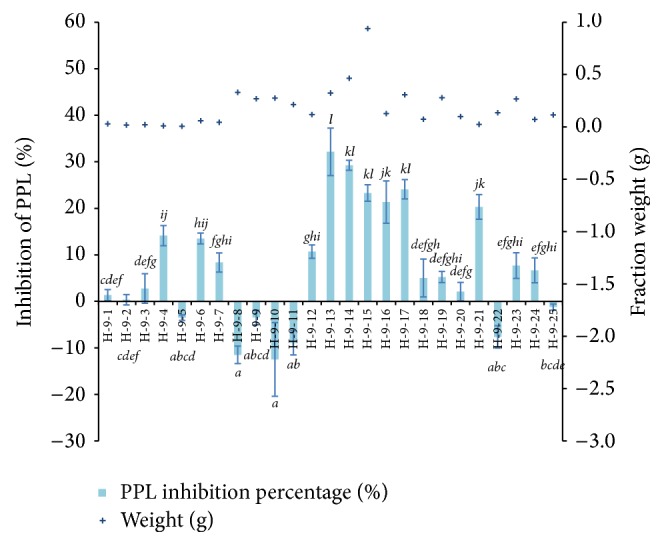
The fraction yield and PPL inhibitory activity of H-9-1 to H-9-25. Bar indicates the standard deviation. Statistically significant effects were compared using one-way ANOVA (Tukey's* post-hoc* test), where different letters indicated significance at *p* < 0.05.

Fraction H-9 (4.21 g) was subjected to further separation due to its high PPL inhibitory activity and sufficient yield.  Figure 6 shows the fraction yield and PPL inhibitory activity of twenty five (25) subfractions of H-9. Highest PPL inhibitory activity was captured in H-9-13 (32.15 ± 5.11%), followed by H-9-14 (29.26 ± 1.08%), where the yield was 0.32 g and 0.47 g, respectively. Subfractions of H-9-13, H-9-17, and H-9-21 recorded PPL inhibitory activity of more than 20% while other subfractions were either promoted or showed less than 20% PPL inhibition.

**Figure 7:**
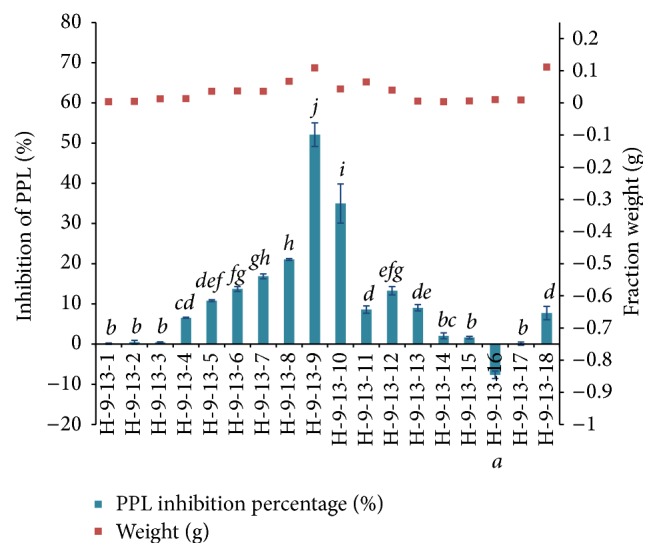
The fraction yield and PPL inhibitory activity of H-9-13-1 to H-9-13-18. Bar indicates the standard deviation. Statistically significant effects were compared using one-way ANOVA (Tukey's* post-hoc* test), where different letters indicated significance at *p* < 0.05.

Both H-9-13 and H-9-14 were selected for further isolation due to their higher yield (323.6 mg and 465.3 mg, resp.). As both H-9-13 and H-9-14 showed comparable PPL inhibition as well as similar TLC spotting profile (data not shown), both subfractions were combined for further isolation and the results of the PPL inhibitory activity of the resultant isolated subfractions are shown in Figure 7.

Highest PPL inhibitory activity was shown by H-9-13-9 (108.8 mg), that is, 52.08 ± 2.93%, which was about 18% higher than that recorded by Orlistat (34.49 ± 5.39%). Fraction H-9-13-10 recorded the second highest PPL inhibition, 34.96 ± 4.87%, which was similar to that of Orlistat.

The TLC profile from H-9-13-9 showed one prominent yellow spot where further isolation was then done, resulting in the isolation of lutein (**3**) (10.8 mg) from this fraction. This isolated pure compound (lutein) was subjected to few analysis including melting point analysis, UV, GC-MS (for the determination of molecular weight), FT-IR (determination of functional group), and NMR (structural elucidation). The characterised lutein was found to possess very strong PPL inhibitory activity, that is, 55.98 ± 1.04% at 100 *μ*g/mL (176 *μ*M), which was 21.5% higher than that recorded in Orlistat (34.49 ± 5.39%). To date, this is the first study to reveal the potential of lutein as a strong PPL inhibitory agent.

### 3.4. Isolation of Compounds from* E. indica*

**Figure 8:**
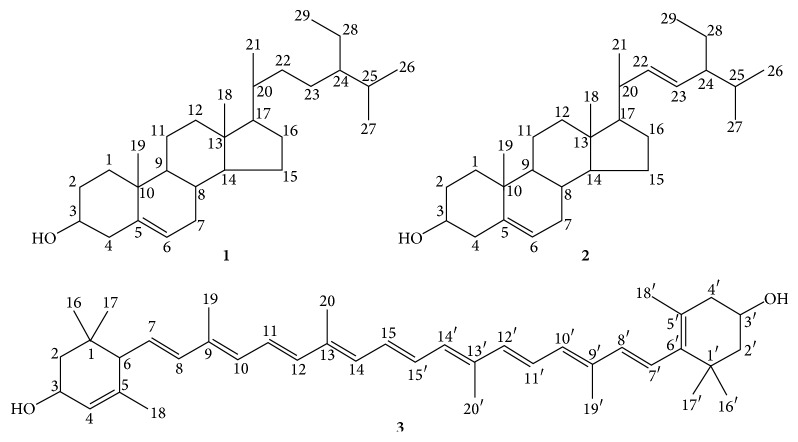
Chemical structures of *β*-sitosterol (**1**), stigmasterol (**2**), and lutein (**3**).

The structures of the isolated compounds were shown in Figure 8. The white crystals of *β*-sitosterol (**1**) had melting point of 134.5–137.6°C [lit. 134-135°C [21]] and also showed IR absorptions at 3431 (O-H stretching), 2937 (aliphatic C-H stretching), 1468 (C-H_2_ bending), 1382 (C-H_3_ bend), and 1056 (=CH bend). EIMS has confirmed the molecular formula C_29_H_50_O with the molecular ion peak at* m/z* 414. Similarly, stigmasterol (**2**) was isolated as white crystals with melting point of 168.0–170.0°C (lit 170°C [22]), and its mass spectral data suggested the molecular formula as C_29_H_48_O. The IR absorption of stigmasterol was similar to that of *β*-sitosterol with an additional absorption band at 1645 cm^−1^indicative of an olefin group. Both *β*-sitosterol and stigmasterol recorded UV absorption maxima at 210 nm. Sterols normally absorb in the range of 190–210 nm, due to the transitions of *π* to *ρ* ^*∗*^ [23].

The ^1^H NMR spectrum of *β*-sitosterol (**1**) had detected multiplet of signals at *δ* 3.21 of H-3 which belonged to the hydroxymethyl group at C-3, and another pair of multiplet resonated at *δ* 4.61 which is assigned to the olefinic proton at H-6. The ^13^C NMR spectrum showed a total of 29 carbon signals were captured, which supported the molecular formula obtained in EIMS. These signals consist of a quaternary methine at C-5 (*δ* 145.3), an olefinic methine at C-6 (*δ* 121.8), as well as several methyl group at C-18, C-19, C-21, C-26, C-27, and C-29 (*δ* 14.9, 18.4, 17.8, 19.6, 18.4, and 15.5, resp.). And methylene group was located at *δ* 39.0 (C-1), *δ* 32.7 (C-2), *δ* 42.1 (C-4), *δ* 31.2 (C-7), *δ* 21.5 (C-11), *δ* 39.5 (C-12), *δ* 26.3 (C-15), *δ* 27.5 (C-16), *δ* 33.4 (C-22), *δ* 26.8 (C-23), and *δ* 25.6 (C-28). Both proton and carbon signals obtained were matched and in agreement with literature [24].

In the ^1^H NMR spectrum of stigmasterol (**2**), there was presence of methyl singlets at *δ* 0.69 (H-18) and *δ* 1.00 (H-19); multiplets at *δ* 5.16 (H-22) and *δ* 5.03 (H-23) signified olefinic protons which were not found in *β*-sitosterol. Similarly, ^13^C NMR had revealed 29 carbon peaks where olefinic carbons were identified at *δ* 140.8 (C-5), *δ* 121.8 (C-6), *δ* 51.3 (C-22), and *δ* 25.5 (C-23); methyl carbons were detected at *δ* 39.8 (C-18), *δ* 19.1 (C-19), *δ* 129.4 (C-21), *δ* 19.5 (C-26), *δ* 19.9 (C-27), and *δ* 12.3 (C-29); methylene carbons were detected at *δ* 37.4 (C-1), *δ* 32.0 (C-2), *δ* 42.4 (C-4), *δ* 31.7 (C-7), *δ* 21.2 (C-11), *δ* 39.9 (C-12), *δ* 24.4 (C-15), *δ* 29.0 (C-16), and *δ* 18.9 (C-28). Both spectra data were matched and in agreement with literature [25].

Lutein (**3**) was isolated as orange crystals with a melting point of 173.8–174.9°C and maxima wavelengths recorded at 426, 448, and 476 nm, which were in agreement with the data of the literature [26, 27]. Mass spectrum of lutein given the molecular ion at* m/z* 568, followed by fragments in* m/z* 550, is attributed to the loss of a hydroxy group. ^1^H NMR spectra data revealed hydroxyl protons resonated at *δ* 4.24 (H-3) and *δ* 3.98 (H-3′); olefinic protons at *δ* 4.24 (H-3), *δ* 5.54 (H-4), *δ* 5.39 (H-7), *δ* 6.10 (H-8), *δ* 6.10 (H-10), *δ* 6.57 (H-11), *δ* 6.34 (H-12), *δ* 6.23 (H-14), *δ* 6.57 (H-15), *δ* 6.10 (H-7′ and H-8′), *δ* 6.57 (H-11′ and H-12′), and *δ* 6.23 (H-14′); allylic protons at *δ* 1.62 (H-18), *δ* 1.90 (H-19), *δ* 1.96 (H-20), *δ* 1.73 (H-18′), and *δ* 1.96 (H-19′ and H-20′); methyl singlets at *δ* 0.838, *δ* 0.987 (H-16 and H-17), and *δ* 1.06 (H-16′ and H-17′). The ^13^C NMR spectra data revealed olefinic carbons resonated at *δ* 125.0 (C-4), *δ* 137.8 (C-5), *δ* 128.8 (C-7), *δ* 138.1 (C-8), *δ* 135.2 (C-8), *δ* 135.2 (C-9), *δ* 130.9 (C-10), *δ* 124.6 (C-11), *δ* 137.6 (C-12), *δ* 136.5 (C-13), *δ* 132.7 (C-14), *δ* 130.1 (C-15), *δ* 126.2 (C-5′), *δ* 137.6 (C-6′), *δ* 125.7 (C-7′), *δ* 138.6 (C-8′), *δ* 135.8 (C-9′), *δ* 131.4 (C-10′), *δ* 124.9 (C-11′), *δ* 137.8 (C-12′), *δ* 136.6 (C-13′), *δ* 132.7 (C-14′), and *δ* 130.2 (C-15′); hydroxyl attached carbon resonated at *δ* 66.0 (C-3) and *δ* 65.2 (C-3′); methyl carbons were detected at *δ* 24.4 (C-16), *δ* 29.6 (C-17), *δ* 22.8 (C-18), *δ* 13.2 (C-19), *δ* 12.8 (C-20), *δ* 28.8 (C-16′), *δ* 30.3 (C-17′), *δ* 21.7 (C-18′), and *δ* 12.9 (C-19′ & C-20′); and methylene carbons resonated at *δ* 44.7 (C-2), *δ* 48.5 (C-2′), and *δ* 42.6 (C-4′). Both ^1^H and ^13^C NMR values were in agreement with published values [26].

Common plant sterols like *β*-sitosterol and stigmasterol are important in cellular and developmental mechanisms in plants [28]. In therapeutic treatment via dietary options, food products supplemented with plant sterols helped in the reduction of plasma cholesterol and the risk of atherosclerosis [29]. However, the cholesterol lowering effect may not be attributed to the inhibition via pancreatic lipase since the PPL inhibition activity of both phytosterols obtained was less than 2%. Weak PPL inhibition activity of *β*-sitosterol and stigmasterol isolated from* Alpinia zerumbet* had also been reported by Chompoo et al. (2012) [30] with IC_50_ value of 99.99 ± 1.86 *μ*g/mL and 125.05 ± 4.76 *μ*g/mL, respectively, in comparison with the inhibition shown by curcumin (IC_50_ = 4.92 ± 0.21 *μ*g/mL) and quercetin (IC_50_ = 18.60 ± 0.86 *μ*g/mL) which were used as positive controls in their study. In our study, *β*-sitosterol and stigmasterol were recorded with weak PPL inhibitory activity of only 3.0 ± 0.8% and 2.7 ± 0.4% at 100 *μ*g/mL, respectively, (i.e., 242 *μ*M and 243 *μ*M) in contrast with that obtained from Orlistat (34.5 ± 5.4% at 100 *μ*g/mL), which were comparatively lower than that recorded in literature (i.e., 50% PPL inhibition at 100 *μ*g/mL) [30]. This may be due to different experimental settings where concentrations of both subtrate and enzyme concentrations used were different in both studies.

Lutein, a member under the xanthophyll family, is the principal carotenoid in greens, leaves, and yellow flowers. The bioavailability of xanthophyll is highly dependent on the matrix due to its hydrophobicity of the long carbon skeletons, and thus dietary lipids are required to facilitate the dispersion of lutein, where similar matrix was adapted by pancreatic lipase [31]. Another study demonstrated xanthophyll ester hydrolysed by cholesteryl esterase, but not triacylglycerol lipase (pancreatic lipase), and thus ruled out the possibility of xanthophyll as a competitive substrate [32, 33]. To date, the mechanism and the inhibitory effect of xanthophyll on pancreatic lipase are still largely unknown, based on the literature findings that may lead to postulation that lutein may be a noncompetitive inhibitor by binding on the allosteric site of pancreatic lipase. Further study is thus required to fully understand the kinetic interactions between lutein and pancreatic lipase.

## 4. Conclusions

Bioactivity-guided isolation on hexane extract of* E. indica* has led to isolation and elucidation of potent PPL inhibitory agent lutein (**3**) with strong inhibitory activity of 55.98 ± 1.04%, alongside with the isolation of two other common sterols: (**1**) *β*-sitosterol and (**2**) stigmasterol. To date, this is the first report on the pancreatic lipase inhibitory activity of lutein.

## Figures and Tables

**Table 1:** Kinetic analyses of PPL inhibition by methanolic crude extract of *E. indica*.

	Velocity of enzyme activity in different concentration of substrate [*S*] (*μ*M)				*V*_max_ (*μ*M min^−1^)	*K*_m_ (*μ*M)
	20	40	60	80		
Control	0.035 ± 0.003	0.026 ± 0.005	0.022 ± 0.005	0.019 ± 0.003	65.79	26.36
100 *μ*g/mL of methanolic *E. indica*	0.043 ± 0.003	0.029 ± 0.001	0.024 ± 0.002	0.023 ± 0.004	63.29	33.70
200 *μ*g/mL of methanolic *E. indica*	0.047 ± 0.005	0.031 ± 0.001	0.029 ± 0.004	0.024 ± 0.003	57.14	34.11

Values are expressed as mean ± SD.

**Table 2:** Yield and PPL inhibitory activity of *E. indica* crude methanolic extract and solvent fractions.

	Yield (g/100 g crude extract)	PPL inhibition (%)
Crude methanolic extract	—	25.05 ± 3.58^c^
Hexane fraction	34.16 ± 7.53	27.01 ± 5.68^c^
Dichloromethane fraction	0.98 ± 0.47	25.57 ± 3.26^c^
Ethyl acetate fraction	1.40 ± 0.47	10.38 ± 2.73^b^
Butanol fraction	9.72 ± 2.36	5.23 ± 2.29^a^
Aqueous fraction	52.78 ± 4.37	4.18 ± 1.91^a^
Orlistat	—	34.49 ± 5.39^d^

Values are expressed as mean ± SD, *n* = 3 values. The concentration of the tested extract against PPL was 100 *μ*g/mL. Statistically significant effects were compared using one-way ANOVA (Tukey's *post-hoc* test), where different letters in superscripts indicated significance at *p* < 0.05.

## Abbreviations

EI: * Eleusine indica*
PPL: Porcine pancreatic lipase
HPLC: High performance liquid chromatography
IR: Infrared
NMR: Nuclear magnetic resonance.
